# Single-port robot assisted concomitant hemi-nephrectomy, ureterectomy and radical prostatectomy using the da vinci SP platform

**DOI:** 10.1016/j.eucr.2020.101550

**Published:** 2020-12-28

**Authors:** Mahmoud Khalil, Alexander Cranwell, John Ouyang, Zaheer Alam, Jean Joseph

**Affiliations:** Department of Urology, University of Rochester Medical Center, Rochester, NY, USA

**Keywords:** Single port, Heminephrectomy, Ureterectomy, Robot-assisted prostatectomy, Radical prostatectomy

## Abstract

A duplicated collecting system is a common congenital anomaly of the urinary tract. However, late symptomatic presentation in adulthood is uncommon. We report the first case of left heminephrectomy, ureterectomy and radical prostatectomy using the da Vinci SP (single port) surgical system in a 64-year-old patient with localized prostate cancer and duplicated system with ectopic ureteral insertion into the prostatic urethra. The procedure was completed without technical difficulties or intraoperative adverse events. We demonstrate that the da Vinci SP robot allows for efficient performance of concomitant surgeries on the kidney and prostate without the limitations reported with single site surgeries.

## Highlights

• A duplicated collecting system is a common congenital anomaly of the urinary tract.
• Late symptomatic presentation of duplicated system in adulthood is uncommon.
• We report single port robotic heminephrectomy, ureterectomy and radical prostatectomy.
• The procedure was completed without technical difficulties or intraoperative events.

## Introduction

Renal duplication is a common congenital anomaly, and is often associated with other pathological conditions such as vesicoureteral reflux, ureterocele, ectopic ureteral insertion, and ureteropelvic junction obstruction. However, up to 20% of patients remain asymptomatic until adulthood.^1^ In the setting of an ectopic ureter in men, all extravesical insertions are suprasphincteric with the prostatic urethra (50%) and seminal vesicle (33%) being the most commonly reported insertion sites.^2^ Herein we describe the first reported concomitant heminephrectomy, ureterectomy, and radical prostatectomy using the da Vinci SP® (single port) surgical system (Intuitive Surgical, Sunnyvale, CA) in an adult with a duplicated system and prostate cancer.

## Case presentation

**Fig. 1:**
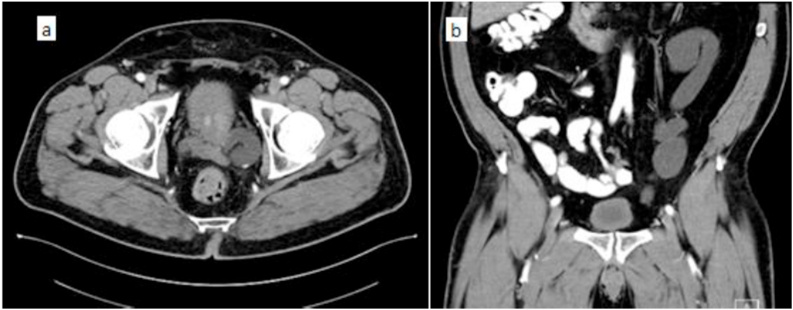
Preoperative CT scan: (a) axial image showing dilated left ectopic ureter twisting behind the prostate and (b) coronal image showing left upper pole hydroureteronephrosis.

A 64-year-old Caucasian man presented with elevated serum prostate-specific antigen (PSA) of 5.4 ng/ml and lower urinary tract symptoms. Transrectal ultrasound-guided biopsy revealed Gleason Score 6 (3 + 3) prostate adenocarcinoma in left apex, mid, base and right apex, and Gleason Score 7 (3 + 4) in right mid, base and left mid gland. Computed Tomography (CT) abdomen and pelvis (Fig. 1) revealed a duplicated left collecting system with severe hydronephrosis of the upper left renal moiety, and hydroureter down to the level of the urinary bladder. The left ureter was tortuous, with an anomalous insertion into the prostatic urethra. A renal scan showed differential function of 54% and 46% in the left and right side respectively. After informed consent, the patient elected to undergo a single port robot-assisted radical prostatectomy, bilateral pelvic lymph node dissection, and left heminephrectomy with upper pole ureterectomy.

**Fig. 2:**
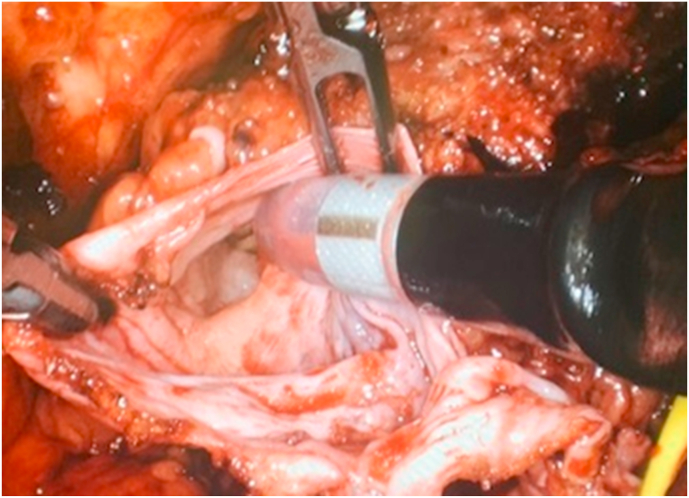
Intraoperative picture showing the lumen of transected distal ectopic ureter at the level of pelvic brim.

Flexible cystoscopy was first performed to place a 5-Fr open end catheter in the normal lower pole ureter. The ectopic ureteral orifice was not visualized. A 4-cm incision was made in the supraumbilical crease, with subsequent placement of a GelPOINT® (Applied Medical, Rancho Santa Margarita, CA). The 2.5-cm SP cannula was inserted through the gel. A 12 mm port was placed in the right lower abdomen. With the patient placed in a mild right lateral position, the left upper abdomen was first approached. The colon was mobilized medially exposing the anterior aspect of the kidney. The normal appearing catheterized lower pole ureter was identified. The ectopic ureter was dissected cephalad with excision of the entire dilated upper moiety. The attached ureter was later traced into the pelvis caudally below the pelvic brim where it was transected (Fig. 2).

**Fig. 3:**
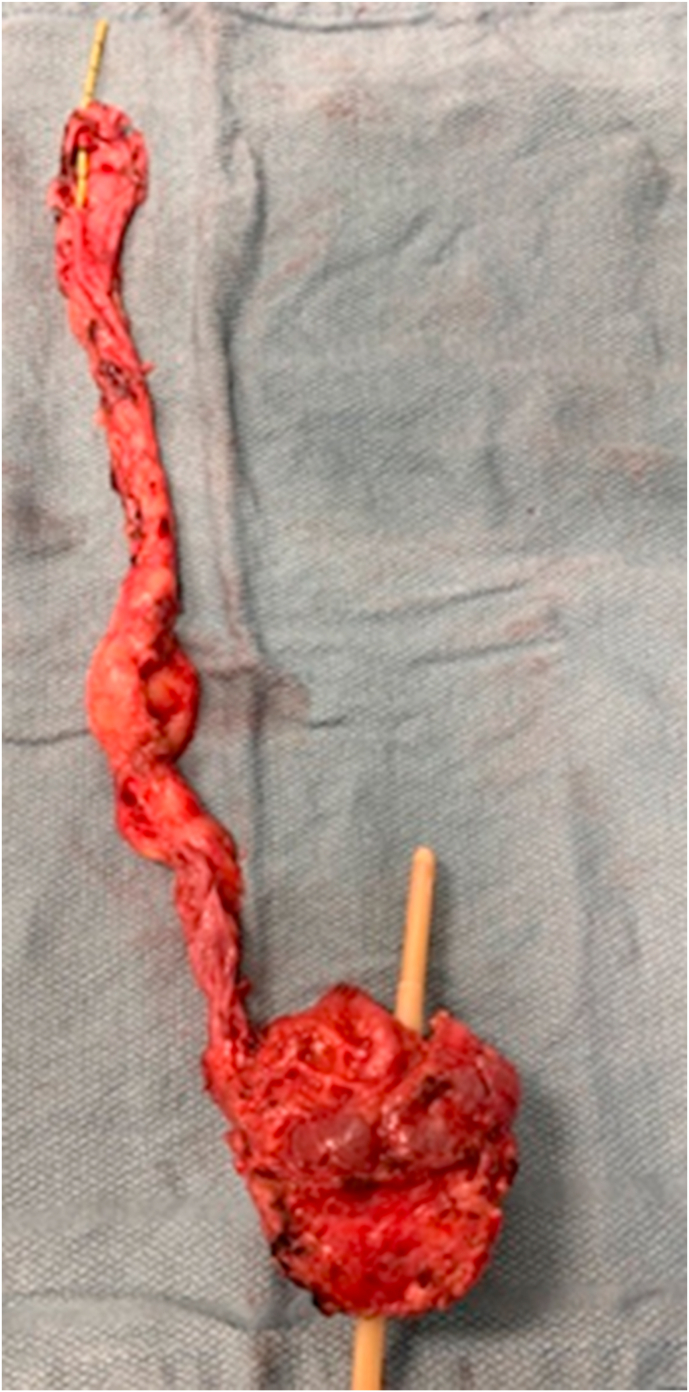
Posterior view of the excised specimen: Ectopic ureter inserted into the prostatic urethra.

At this point, the SP robot was re-directed to the pelvis for the planned radical prostatectomy. With the operating table positioned at 15° Trendelenburg, the bladder was dissected off the abdominal wall allowing entry into the Retzius space. The endopelvic fascia was incised and the dorsal vein bundle was controlled using a Covidien V-LocTM (Covidien, New Haven, CT) suture on an SH needle. The bladder neck was transected with subsequent dissection of the seminal vesicles. The dilated left upper pole ureter was noted bulging along the left pelvis beneath Denonvilliers' fascia. The latter was entered exposing the anterior surface of the rectum. The left ectopic ureter was identified as it enters the prostate. Its cephalad portion was dissected behind the bladder and pulled from the rectovesical space leaving it attached to the prostate posteriorly (Fig. 3). The prostate apex was dissected with transection of the dorsal vein and urethra. The urethrovesical anastomosis was performed. The peritoneum overlying the bladder was anchored laterally to the bladder wall to maintain continuity of the peritoneal surface with the lymphadenectomy fossa. A Jackson-Pratt drain was placed in the space of Retzius. The specimens previously placed in an EndoCatch bag were retrieved through the fascia opening, which was later closed using 0-vicryl sutures. Estimated blood loss was 200 cc.

The post-operative course was uneventful, except for a mild ileus. Hospital length of stay was 4 days. JP drain was removed prior to discharge. The Foley catheter was removed a week post-surgery. Final pathology was Stage T3aN0, with extraprostatic extension on the right side, Gleason Score 4 + 3. All lymph nodes and surgical margins were negative.

## Discussion

The development of novel surgical instruments has led to increased adoption of minimally invasive surgery which has been associated with improved outcomes, decreased postoperative pain, and lower complication rates compared to open surgery.^3^ With evolution of this trend, the SP surgical system has been increasingly used to perform major urological procedures, namely robot-assisted radical prostatectomies (RARP), robot-assisted radical and partial nephrectomies, and various ureteral reconstructive surgeries.^4^

Upper tract renal surgeries using SP surgical system have been shown to be safe with promising results. Similarly, SP lower tract urologic surgeries have been reported, and are becoming routine at several institutions. Our report is the first to demonstrate the effectiveness of the SP surgical system in completing a heminephrectomy, ureterectomy, prostatectomy, and bilateral pelvic lymphadenectomy. With the relocation pedal, the arms are moved to the desired quadrant effectively without the need for undocking. In our patient, we had placed him in a mild right lateral decubitus position to facilitate retraction of the bowel with gravity. To access the pelvis, Trendelenburg position was required to keep the bowel out of the operative field. The robot was therefore undocked after the renal portion to reposition the patient. The table and robot pairing is not currently available for the SP robot. Therefore, undocking is necessary if change in the patient position is required. No instrument limitations were encountered. Similarly, no complication was encountered during the radical prostatectomy and lymphadenectomy portion.

Kaouk et al. first reported SP-RARP in two patients without recorded complications.^4^ This was followed by several studies that showed the safety and feasibility of a RARP using the SP.^5^ SP-RARP has been demonstrated in both anterior and posterior approaches. Transperitoneal and extraperitoneal approaches have also been described. We used a transperitoneal approach in the present case, given the concomitant heminephrectomy and ureterectomy.

## Conclusion

The SP robot allows easy access to the upper retroperitoneum and pelvis for multi-organ surgeries. Although SP surgical system resolves many limitations associated with single site surgery, the overall value to our surgical armamentarium will be determined further with increased utilization or adoption by experienced centers.

## Consent

The patient provided a consent for this case report and agreed to the publication of details and figures related to the case.

## Funding sources

This research did not receive any specific grant from funding agencies in the public, commercial, or not-for-profit sectors.

## Author statement

**Mahmoud Khalil:** Conceptualization; Data curation; Resources; Writing - original draft.

**Alexander Cranwell:** Conceptualization; Validation; Writing - original draft.

**John Ouyang:** Conceptualization; Resources; Software; Writing - review & editing.

**Zaheer Alam:** Supervision; Validation; Writing - review & editing.

**Jean Joseph:** Conceptualization; Supervision; Validation; Writing - review & editing.

## Declaration of competing interest

JJ is a consultant with Boston scientific. All other coauthors report no conflicts of interest.

## References

[1] Ellerker A.G. (1958). The extravesical ectopic ureter. Br J Surg.

[2] Didier R.A., Chow J.S., Kwatra N.S., Retik A.B., Lebowitz R.L. (2017). The duplicated collecting system of the urinary tract: embryology, imaging appearances and clinical considerations. Pediatr Radiol.

[3] Rassweiler J.J., Teber D. (2016). Advances in laparoscopic surgery in urology. Nat Rev Urol.

[4] Kaouk J., Garisto J., Eltemamy M., Bertolo R. (2019). Pure single-site robot-assisted partial nephrectomy using the SP surgical system: initial clinical experience. Urology.

[5] Jones R., Dobbs R.W., Halgrimson W.R. (2020). Single port robotic radical prostatectomy with the da Vinci SP platform: a step by step approach. Can J Urol.

